# Face Recognition by Metropolitan Police Super-Recognisers

**DOI:** 10.1371/journal.pone.0150036

**Published:** 2016-02-26

**Authors:** David J. Robertson, Eilidh Noyes, Andrew J. Dowsett, Rob Jenkins, A. Mike Burton

**Affiliations:** Department of Psychology, University of York, York, United Kingdom; University of Lincoln, UNITED KINGDOM

## Abstract

Face recognition is used to prove identity across a wide variety of settings. Despite this, research consistently shows that people are typically rather poor at matching faces to photos. Some professional groups, such as police and passport officers, have been shown to perform just as poorly as the general public on standard tests of face recognition. However, face recognition skills are subject to wide individual variation, with some people showing exceptional ability—a group that has come to be known as ‘super-recognisers’. The Metropolitan Police Force (London) recruits ‘super-recognisers’ from within its ranks, for deployment on various identification tasks. Here we test four working super-recognisers from within this police force, and ask whether they are really able to perform at levels above control groups. We consistently find that the police ‘super-recognisers’ perform at well above normal levels on tests of unfamiliar and familiar face matching, with degraded as well as high quality images. Recruiting employees with high levels of skill in these areas, and allocating them to relevant tasks, is an efficient way to overcome some of the known difficulties associated with unfamiliar face recognition.

## Introduction

Face recognition is a fundamental part of our everyday lives. We can recognise familiar faces over a very wide range of settings, including visually difficult conditions such as poor lighting or degraded images [1]. However, our ability to recognise familiar people does not generalise well to unfamiliar faces. It has been known for many years that eyewitness identification is highly error-prone (see [2] for a review). More recently, it has become clear that face *matching* is also very difficult for unfamiliar viewers. Even with no requirement to remember anything, viewers find it very difficult to match two photos of the same person even when these are taken on the same day, in good lighting, and when there are no time-restrictions [3–7]. The difficulty of unfamiliar face matching extends into real life settings; in modern society, we are often asked to prove our identity by presenting photo-ID. However, viewers find it very difficult to match a live face to a photo or video, making many errors [8–10].

Face recognition, and particularly unfamiliar face matching, is also a key part of forensic and security operations. To cross national borders we often have to present a passport (with photo), and if we are ever accused of a crime, CCTV evidence may be used. The frequency of this task, combined with its difficulty, raises the question of whether professionals comparing face images (e.g. passport officers, the police or security agencies) can do so effectively. While there has been movement towards automation of facial recognition in some settings, automatic face recognition is neither universal nor infallible, and human operators almost always have the final say in identification judgements [11]. The face recognition accuracy of trained professionals is therefore critical to the accuracy of the system as a whole.

The results of studies on face experts have been somewhat mixed. White et al [12] tested a group of 49 passport issuing officers and demonstrated that they were no better on standard measures of face matching than a group of untrained students. Similarly, Burton et al [1] showed that a group of police officers were no better than untrained students at matching poor quality CCTV images to facial photos. Furthermore, the work of forensic experts, providing specialist evidence on face matching to courts has been challenged as unreliable [13–14]. More recently, some studies of professionals specifically trained to make forensic image comparisons have demonstrated that they can out-perform untrained students [15–16]. In the case of passport officers, a sub-group with particular responsibility for facial comparison performed better than other officers [11].

Interestingly, any differences that are observed between professional and novice groups are typically small by comparison to within-group differences. For example, in the study on passport officers [12], performance on a live-face-to-photo matching task ranged between 100% and 70% within the group (where chance level was 50%). Indeed there is growing appreciation of the large individual variation in face recognition abilities within the population [17–18]. Furthermore, the extreme ends of the distribution of face recognition ability have drawn interest in their own right. *Congenital prosopagnosia* refers to the phenomenon by which some members of the general population are extremely bad at face recognition (in the clinical range) despite having no known pathology [19–20]. At the top end of the distribution are *super-recognisers* who display very high levels of ability on a range of face recognition tasks [21].

Importantly for applied face recognition, these very large individual differences appear to be highly stable over time. Among passport officers, White et al [12] found no correlation between years of service (range 0–21 years) and task performance (range 58–95%), implying that occupational experience does nothing to improve accuracy. Moreover, the lack of any group difference between the passport officers, who had received professional training in facial image comparison, and undergraduate students, who had not, implies that the occupational training had no effect on accuracy either. The absence of any training benefit in this context is consistent with laboratory studies, where generalizable training benefits have typically been small or absent [22–24].

Given the scale of the individual differences in face recognition ability, and the limited impact of experience and training, it seems natural to propose *personnel selection* as an approach to optimising performance: simply recruiting individuals with a talent for face recognition should improve the accuracy of the system [12]. To this end, the Metropolitan Police, the force responsible for London, have recently set up a ‘super-recogniser team’ [25]. Members of the team are recruited from within the police force, on the basis of a particular interest in face recognition, and an undisclosed facial memory test. They perform a wide variety of tasks, including identification of suspects live and from security cameras. They often work with low-quality images and with people who are attempting to hide their identity.

In this study we ask a simple question: are the police super-recognisers actually better than the normal population on tests of face recognition? Since they give evidence in court based on their expertise, and the recognition decisions they make are (by definition) hard for other viewers to confirm, we sought to test their ability over several face recognition tasks, including standardised tasks and a comparison to regular police officers. To anticipate the findings, we did establish that the ‘super-recognisers’ we tested had face recognition skills which far exceed the general population.

## Method and Results

### Ethics Statement

**Fig 1:**
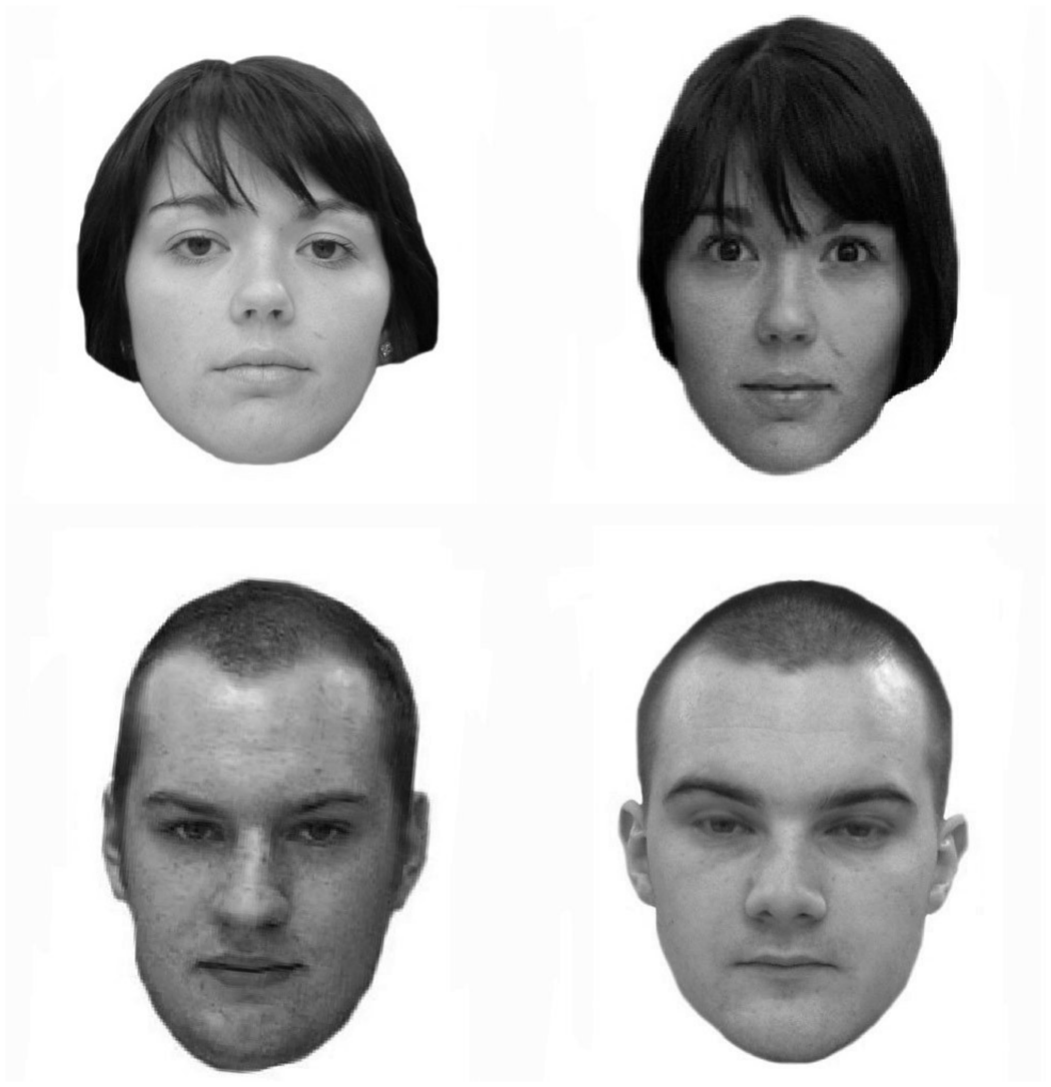
Example trials from the Glasgow Face Matching Test (GFMT). The top pair shows two instances of the same person, the bottom pair shows two different people. The individuals shown in Fig 1 have given written informed consent (as outlined in PLOS consent form) to publish these images.

This study was approved by the Ethics Committee of the Department of Psychology, University of York. All participants gave written informed consent. The individuals shown in Fig 1 have given written informed consent (as outlined in PLOS consent form) to publish these images.

### Participants and Testing

We studied four Metropolitan Police staff who were part of the super-recogniser (SR) team at New Scotland Yard Central Forensic Image Unit, London, at the time of testing (summer 2015). All were male, and the mean age was 40 (range 33–47). The SRs were tested during a normal working day, in a shared office—their usual working environment.

We carried out three tests of face recognition. 1) The Glasgow Face Matching Test (GFMT) [26], which is a standardised test of unfamiliar face matching; 2) an unfamiliar face matching test that was designed to be particularly difficult, the Models Face Matching Test (MFMT) [27]; and 3) the Pixelated Lookalikes Test (PLT)—a new test of *familiar* face recognition based on very poor quality images.

### Task 1: Glasgow Face Matching Test

#### Test

The GFMT (short version) consists of 40 pairs of unfamiliar faces, half of which are same identity pairs and half of which are different identity pairs (see Fig 1 for examples). Each face image is front facing, neutral in emotional expression, and standardised to a width of 350 pixels (greyscale, 72ppi resolution). Photos were taken a few minutes apart using different cameras. Fig 1 shows examples from the test. For more details see [26].

#### Procedure

Printed paper booklets were used to present the face pairs (one pair per page). Participants were instructed to decide whether the face pairs depicted the same person or two different people. They were required to make their responses by circling the word ‘SAME’ or ‘DIFFERENT’ printed in bold text below the face images. Same face pairs and different face pairs were randomly intermixed. No time constraints were placed on the participants, with individuals working through the booklet at their own pace.

#### Comparison Group

Super-recogniser performance on the GFMT was compared with data from 194 police trainees from a UK training college, who had been used to provide original norms for this version of the test [26]. This was a younger group, on average, than the super-recognisers (mean 26 years, range 18–46), though previous research has demonstrated no association between age and performance on this test [26].

#### Results

**Fig 2:**
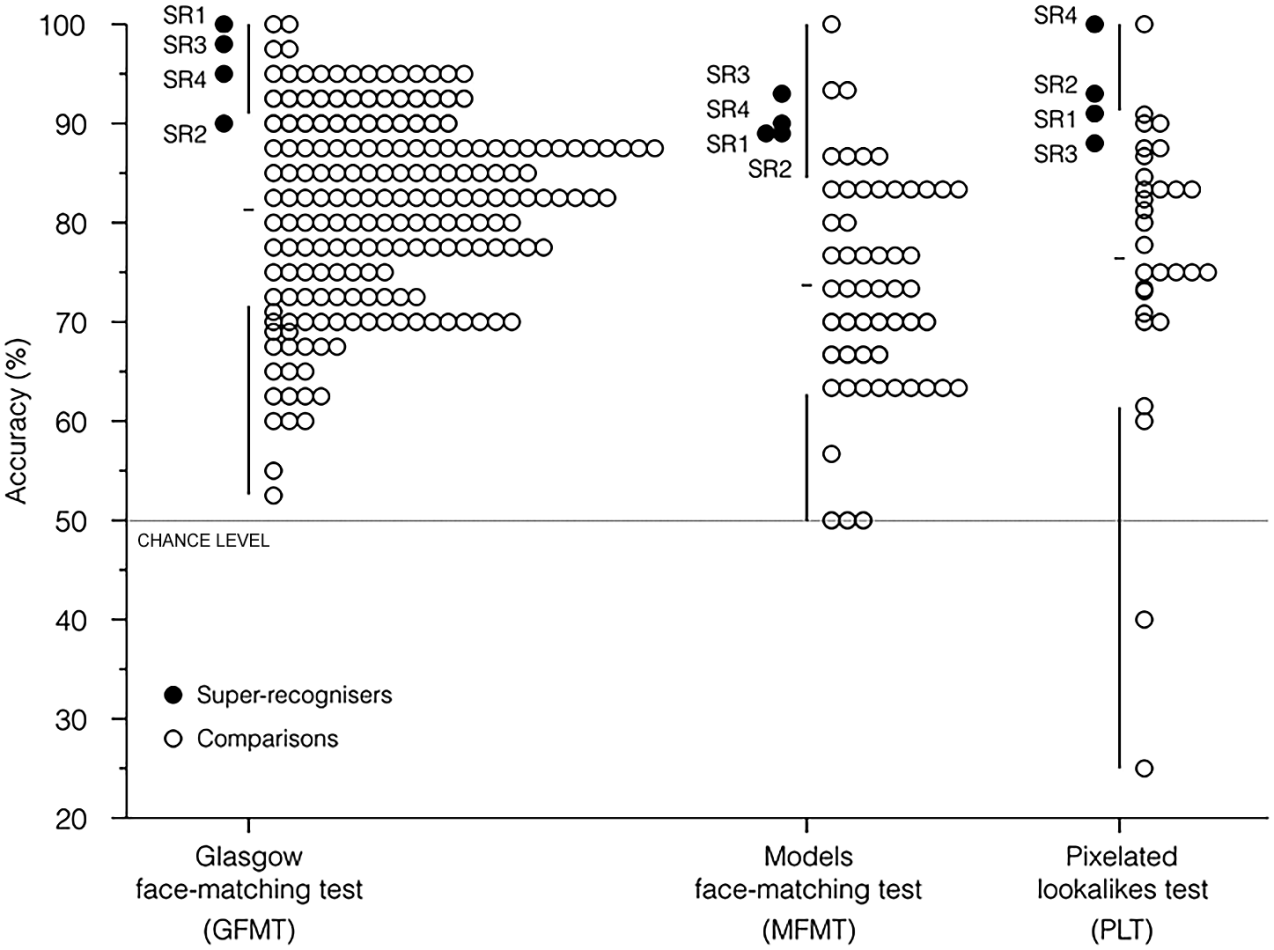
Performance of police super-recognisers and comparison viewers. Performance of super-recognisers (SR1–4; black) and comparison viewers (white) on three different tests of face recognition—the GFMT (left column), the MFMT (middle column), and the PLT (right column). Vertical lines indicate the range of scores for comparison groups, the deleted portion of the line shows the standard deviation, and the horizontal notch shows the mean. In all three tasks, chance performance is 50%.

The super-recognisers were found to outperform a large group of police trainees. While mean accuracy on this test, as derived from the normative police trainee data, is 81.3% (SD = 9.4%), mean accuracy in the super-recogniser group was found to be 95.8% (SD = 4.3%). Fig 2 shows individual scores for each of the SRs, demonstrating very high levels of performance, including one participant scoring 100% accuracy on this test.

### Matching Task 2: Models Face Matching Test

#### Test

The Models Face Matching Test (MFMT, [27]) is a paired face-matching test with the same format as the GFMT, but designed to be more difficult. It consists of 90 pairs of faces, half of which show the same identity, and half of which show different identities. The images depict male models, and were collected from a single portfolio website for professional models. The models carry out photo-shoots with wide variation in clothes, hairstyles, lighting conditions and cameras. For this test, images were presented in full colour and were cropped to display the face only and were standardised to a size of 300 x 420 pixels (for more details see [27]; example images cannot be reproduced here for copyright reasons).

#### Procedure

The procedure for the models matching task was identical to that descried for the GFMT (Matching Task 1) above. Test items were presented in a printed booklet, and participants were asked to make same/different judgements for each pair, with no time limits.

#### Comparison Group

On this task, super-recogniser performance was compared to a normative control group reported in [27]. Participants were 54 undergraduate university students (25 male) with a mean age of 23 years (SD = 4 years, range = 18–40 years).

#### Results

Fig 2 shows performance for super-recognisers and controls. Performance was lower overall than for the GFMT, confirming that this is a harder test, as designed. More strikingly, the super-recognisers once again consistently out-performed the controls. The mean accuracy for the control group, derived from university undergraduates, was 73.6% (SD = 10.9%). Mean accuracy for the super-recognisers was considerably higher at 90.3% (SD = 1.9%), and there was consistently high performance across this group.

### Matching Task 3: Pixelated Lookalike Test

We have seen that the police super recognisers consistently outperform control groups on two different tests of unfamiliar face matching. For the final test, we consider familiar face recognition. As this arises in some policing contexts (for example recognition of previous offenders in security video), it is important to establish whether the high levels of performance we have reported so far extend to the familiar case. Because familiar face recognition is generally so accurate [1], we developed a test that makes it more challenging than normal. The test materials are pixelated images of celebrities and celebrity lookalikes, designed to emulate the poor quality images which are often reviewed in forensic practice.

#### Test

The pixelated lookalike test (PLT) was designed to be presented in the same way as the unfamiliar tests above, i.e. as a same/different matching pairs task. We chose 30 famous celebrities, and selected three images for each of these people from an internet search. One criterion for selection was the ready availability of professional lookalikes for these celebrities, and we chose one representative image of a lookalike for each celebrity. We were therefore able to create two trials per celebrity: a *match* trial comprising two images of that person, and a *mismatch* trial comprising an image of the celebrity and an image of the corresponding lookalike. Each image was only used once across match or mismatch trials.

**Fig 3:**
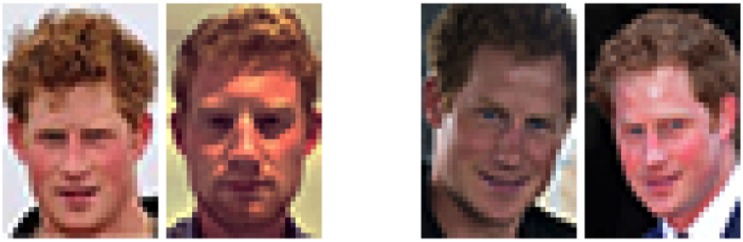
Example trials from the PLT. Images on the left show different identities (with the imposter face on the right). Images on the right show the same identity.

To emulate poor quality security surveillance we presented all images in low resolution (30 pixels wide x 45 pixels high). This also served the purpose of reducing matching images to the level that we expect to avoid ceiling effects in the *matching* trials. Examples of the stimuli are shown in Fig 3.

#### Procedure

The procedure was similar to that used in the unfamiliar tests. Super-recognisers were presented with a printed booklet containing 60 trials, half of which showed the same identity and half of which showed different identities. They were asked to make a same/different judgement for each trial. We were keen to ensure that this was genuinely a test of familiar face matching, and so following the face-matching task participants used a numerical scale to indicate their level of familiarity with each celebrity whose face had been viewed in the task (from 1 [completely unfamiliar] to 10 [extremely familiar]).

#### Comparison Group

30 students (11 male) were recruited from the University of York to act as a comparison group for this study. Mean age was 19.7 (range 18–28, SD = 2.8). All took part in return for course credit. As the comparison group were generally younger than the super-recognisers, we took care to ensure that any differences in performance could not arise through some celebrities being more familiar to one group or the other, see below.

#### Results

We are concerned here primarily with familiar face matching, and so we considered only those faces which participants rated as highly familiar in the post-test (8–10 on the 10-point scale). Fig 2 shows the overall pattern of performance. Once again, the police super-recognisers consistently performed with far greater accuracy than student participants. Overall, the controls scored just 73% correct, whereas the police super-recognisers scored with 93% accuracy in the lookalike test. All super-recognisers performed much better than the control mean, with one super-recogniser performing perfectly.

The preceding analysis was based on the subset of faces that viewers rated as highly familiar. Interestingly, super-recognisers gave high familiarity ratings (8–10) to a very high proportion of faces compared with controls (70% of faces for super-recognisers; 37% of faces for controls). To better understand the super-recogniser advantage, we conducted two further analyses. First, we compared accuracy of super-recognisers and controls on faces that they rated as less familiar (0–7 on the 10-point scale; i.e. those not included in the above analysis). Police super-recognisers outperformed controls on these faces too (80% accuracy for super-recognisers; 67% accuracy for controls), implying that their performance advantage holds across the whole familiarity continuum. Second, we analysed the control participants’ data for an association between (i) the proportion of faces that were given high familiarity ratings and (ii) the level of accuracy on those highly familiar faces. We found a significant positive correlation between these two measures [r(28) = 0.39, p < .05], such that the highest performing controls were qualitatively similar to the super-recognisers. These analyses support the original comparison, i.e. our SRs are performing at well above the levels of controls, even when group differences in famous face familiarity are taken into account.

## Discussion

We have now seen consistently high performance of the police super-recognisers across three different tasks. Interestingly, their strong performance on the tests of unfamiliar face matching (GFMT and MFMT), are accompanied by strong performance on familiar face matching too (PLT). These are all components of everyday policing, but familiar and unfamiliar face processing abilities are not always reported to be associated [17].

The selection criteria for the Metropolitan Police super recogniser team are not made public—as one might expect. However, they appear to be successful, in that the SRs tested here all performed at well-above normal levels. Furthermore, in the case where we have police controls, the SRs performed well above these too. There is also accumulating evidence that people who self-identify as super-recognisers in the general population perform well on forensically-relevant tasks similar to those used here [28].

It is beginning to be clear that simple membership of a professional group dealing with faces is not sufficient to confer high levels of performance [1,12]. For example, previous work with police officers and passport officers shows that, on average, both professional groups perform face tasks with the same levels as the general public. However, the wide levels of individual variation in the general population mean that large organisations have the opportunity to sample from within their ranks, and allocate those with appropriate skills to relevant jobs. The evidence to date suggests that training for unfamiliar face recognition is typically not highly effective. Recruitment is therefore key to improving overall levels of performance. The tests reported here provide a consistent indicator of expertise and are quick and easy to administer. We therefore suggest that they may be useful in future recruitment for other agencies. All three tests are available from the authors.

## Supporting Information

S1 Table Scores for super recognisers and comparison participants on each of the three tests. (XLSX)

S1 Text Figure permissions. (DOCX)
