# Supplementary material for: Face Recognition by Metropolitan Police Super-Recognisers
Source: PLoS One. 2016 Feb 26;11(2):e0150036. doi: 10.1371/journal.pone.0150036 (PMC4769018; doi:10.1371/journal.pone.0150036)
Supplement: S1 Text — (DOCX) [file pone.0150036.s002.docx]

Fig 1 Copyright A.M. Burton (corresponding author), free to publish under the Creative Commons Attribution License (CCAL), CC BY 4.0.

Fig 3 Left to right

1

Source: https://commons.wikimedia.org/wiki/File:Harry_Wales_2008.jpg

Photo credit: Billpolo

License: Creative Commons Attribution-Share Alike 3.0 Unported

2

Source: Henry Morley

Photo credit: Henry Morley

License: Creative Commons Attribution License CC BY4.0.

3

Source: <https://en.wikipedia.org/wiki/Prince_Harry#/media/File:Prince_Harry_Invictus_2014.jpg>

Photo credit: U.S. Air Force photo/Staff Sgt. Andrew Lee

License: Public Domain

4

Source: https://commons.wikimedia.org/wiki/File:Prince_Harry_June_2014.jpg

Photo credit: See Li

License: Creative Commons Attribution 2.0 Generic
